# Anti-Trypanosomal Proteasome Inhibitors Cure Hemolymphatic and Meningoencephalic Murine Infection Models of African Trypanosomiasis

**DOI:** 10.3390/tropicalmed5010028

**Published:** 2020-02-17

**Authors:** Srinivasa P S Rao, Suresh B Lakshminarayana, Jan Jiricek, Marcel Kaiser, Ryan Ritchie, Elmarie Myburgh, Frantisek Supek, Tove Tuntland, Advait Nagle, Valentina Molteni, Pascal Mäser, Jeremy C Mottram, Michael P Barrett, Thierry T Diagana

**Affiliations:** 1 Novartis Institute for Tropical Diseases, 5300 Chiron Way, Emeryville, CA 94608, USA; suresh.b_lakshminarayana@novartis.com (S.B.L.); jan.jiricek@novartis.com (J.J.); thierry.diagana@novartis.com (T.T.D.); 2 Swiss Tropical and Public Health Institute, Socinstrasse 57, 4501 Basel, Switzerland; marcel.kaiser@swisstph.ch (M.K.); pascal.maeser@swisstph.ch (P.M.); 3 Department of Epidemiology and Public Health, University of Basel, Petersplatz 1, 4000 Basel, Switzerland; 4 Wellcome Centre for Integrative Parasitology, Institute of Infection, Immunity and Inflammation, College of Medical, Veterinary and Life Sciences, University of Glasgow, Glasgow G12 8TA, UK; Ryan.Ritchie@glasgow.ac.uk (R.R.); Michael.Barrett@glasgow.ac.uk (M.P.B.); 5 York Biomedical Research Institute, Hull York Medical School, University of York, Wentworth Way, Heslington, York YO10 5DD, UK; elmarie.myburgh@york.ac.uk; 6 Genomics Institute of the Novartis Research Foundation, 10675 John Jay Hopkins Drive, San Diego, CA 92121, USA; fsupek@gnf.org (F.S.); tovetuntland@outlook.com (T.T.); anagle@gnf.org (A.N.); vmolteni@gnf.org (V.M.); 7 York Biomedical Research Institute, Department of Biology, University of York, Wentworth Way, Heslington, York YO10 5DD, UK; jeremy.mottram@york.ac.uk

**Keywords:** sleeping sickness, drug discovery, *Trypanosoma* growth inhibitors

## Abstract

Current anti-trypanosomal therapies suffer from problems of longer treatment duration, toxicity and inadequate efficacy, hence there is a need for safer, more efficacious and ‘easy to use’ oral drugs. Previously, we reported the discovery of the triazolopyrimidine (TP) class as selective kinetoplastid proteasome inhibitors with in vivo efficacy in mouse models of leishmaniasis, Chagas Disease and African trypanosomiasis (HAT). For the treatment of HAT, development compounds need to have excellent penetration to the brain to cure the meningoencephalic stage of the disease. Here we describe detailed biological and pharmacological characterization of triazolopyrimidine compounds in HAT specific assays. The TP class of compounds showed single digit nanomolar potency against *Trypanosoma brucei rhodesiense* and *Trypanosoma brucei gambiense* strains. These compounds are trypanocidal with concentration-time dependent kill and achieved relapse-free cure in vitro. Two compounds, GNF6702 and a new analog NITD689, showed favorable in vivo pharmacokinetics and significant brain penetration, which enabled oral dosing. They also achieved complete cure in both hemolymphatic (blood) and meningoencephalic (brain) infection of human African trypanosomiasis mouse models. Mode of action studies on this series confirmed the 20S proteasome as the target in *T. brucei*. These proteasome inhibitors have the potential for further development into promising new treatment for human African trypanosomiasis.

## 1. Introduction

Human African trypanosomiasis (HAT) is a neglected tropical disease caused by the protozoan parasites *Trypanosoma brucei gambiense* and *Trypanosoma brucei rhodesiense*. The disease is endemic to sub-Saharan Africa and transmitted by tsetse flies (*Glossina* spp.). Over the last decade, there has been a significant reduction in the number of new cases of HAT, reaching below ~1000 reported new cases per annum in 2018 [1]. HAT comprises hemolymphatic (stage 1) and meningoencephalic (stage 2) infections. The successful introduction of nifurtimox–eflornithine combination therapy (NECT) for the treatment of gambiense HAT significantly helped in achieving a cure in stage 2 HAT patients [2]. Although NECT is effective, it requires long infusions and continuous monitoring. The introduction of fexinidazole, as an oral drug capable of curing stage 1 and stage 2 disease, offers great potential, and a further orally available drug, acoziborole, is currently being evaluated in late-stage clinical trials [3]. For treatment of rhodesiense HAT, suramin and melarsoprol (a highly toxic arsenical) are still used. New drugs remain desirable if we are to assure no repeat of the historical re-emergence of HAT, following a successful campaign in the mid-twentieth century where cases had dropped to the low thousands, only to resurge to an estimated 300,000 cases by the turn of the century [4]. Recent publications showing that trypanosomes dwell in adipose tissue [5] and skin [6], along with several reports of possible animal reservoirs of gambiense trypanosomes and latent human infections, all point out potential threats to the elimination of HAT [7]. 

Novartis, in collaboration with academic partners, embarked to find novel, safe short-course therapies for treatment of all forms of HAT. Previously, we reported [8] the discovery of the triazolopyrimidine (TP) chemical class as growth inhibitors of *Leishmania donovani*, *Trypanosoma cruzi* and *T. b. brucei* and identified the 20S proteasome as the target responsible for the pharmacological activity. Furthermore, exemplar from this class (GNF6702) demonstrated efficacy in the murine models for the three indications [8]. An earlier study had shown in vitro growth inhibition activity of TP cpds against *T. b. brucei* and GNF6702’s stage 2 efficacy at highest dosing regimen of 100 mg/kg once daily [8]. Here, we describe detailed biological, chemical and pharmacological characterization of the three TP class of inhibitors (GNF3849, GNF6702 and NITD689) in various HAT-specific assays. The TPs are active against disease causing *T. b. gambiense* and *T. b. rhodesiense* strains, as well as drug-resistant (melarsoprol and pentamidine) isolates. These compounds inhibit the chymotrypsin activity of the 20S proteasome and are trypanocidal showing concentration-time dependent kill. Stage 2 HAT treatment requires compounds to have unique properties that enable them to cross the blood–brain barrier in order to be efficacious against CNS-resident parasites. Two compounds, GNF6702 and the newer analog NITD689, had favorable physicochemical and pharmacokinetic properties amenable for oral dosing and achieving free brain concentrations required for stage 2 efficacy. They also achieve relapse-free cure in mouse models of stage 1 and 2 trypanosomiasis, in a dose-dependent manner, suggesting the potential to treat all forms of HAT.

## 2. Materials and Methods

### 2.1. Parasites, Cell Culture and Growth Inhibition Assays

The *T. b. brucei* strain Lister 427 (bloodstream form) parasites were continuously grown in complete HMI-9 medium supplemented with 10% Serum Plus and 10% heat-inactivated fetal bovine serum (FBS) [8]. All other parasite strains were cultured as described elsewhere [9].

For determination of 50% growth inhibition, all compounds were dissolved in DMSO, and 200 nL of threefold serially diluted compounds were added into solid-bottom 384-well white plates (Greiner Bio-One, Kremsmunster, Austria) by an Echo 555 acoustic liquid-handling system. Forty microliters of 10^4^ cells/mL of *T. b. brucei* parasites was added into each well, and the plates were incubated in a 5% CO_2_ incubator at 37 °C for 48 h. Viability of parasites were determined by measuring intracellular ATP levels, using CellTiter-Glo (CTG) luminescent cell viability reagent (Promega, Madison, Wisconsin WI, USA). The EC_50_ values were determined by using GraphPad Prism software. Growth inhibition assays for all clinical isolates were carried out as described earlier [9]. All experiments had two technical replicates and three biological replicates. Appropriate statistical tests were used for evaluating significance, and mean ± SEM is represented in the tables.

### 2.2. HepG2 Cytotoxicity Assay 

The HepG2 (human hepatocellular carcinoma) cells were obtained from ATCC (American Type Culture Collection) and grown in RPMI media. Cytotoxicity assay was performed in 384-well format, and 25 µL, approximately 1.6 × 10^4^ cells/mL, suspension was added to clear-bottom 384-well Griener plates and incubated in 5% CO_2_ incubator, at 37 °C for 24 h. Once the cells adhered, 125 nL of threefold serially diluted compounds in DMSO was added. Plates were incubated for 96 h, at 37 °C, in a 5% CO_2_ incubator. Plates were read for viability by adding 10 µL of CCK-8 reagent (APExBIO) into each well, followed by 3 h incubation and absorbance reading at 450 nM, using an Envision plate reader. Absorbance values were used for determination of cytotoxic concentration (CC_50_) required to inhibit growth by 50%, using GraphPad Prism software. Puromycin was used as a positive control. All experiments had two technical replicates and three biological replicates. Appropriate statistical tests were used for evaluating significance, and mean ± SEM is represented in the tables.

### 2.3. Determination of Solubility, PAMPA, Plasma Protein Binding, Brain Tissue Binding and Microsomal Clearance

Test compounds’ solubility was determined in a high-throughput thermodynamic solubility assay, as described previously [10]. The PAMPA (parallel artificial membrane) assays were carried out, using a standard protocol [11]. Plasma protein binding was determined by using mouse blood [12], whilst brain tissue binding was determined by using rat brain tissue homogenate. Intrinsic metabolic clearances were determined in mouse liver microsomes, using the compound depletion approach and LC–MS/MS quantification [13].

### 2.4. Time to Kill and Reversibility Assays

Time-to-kill experiments were carried out to determine the ability of compounds to kill bloodstream form of *T. b. brucei* Lister 427 at 6, 24 and 48 h post-compound treatment. Viability of parasites were assessed by measuring ATP content as a surrogate. The assay was conducted in 384-well format, in a similar manner as the growth inhibition assay stated above, but with minor modifications. Compound-containing plates were incubated with 40 µL of approximately 1 × 10^5^ parasites per mL, and at each time point, CTG reagent was added to lyse the parasites, and luminescence was measured after 30 min of incubation, using a Tecan M1000 plate reader. 

Reversibility assessment to establish time and concentration required to achieve irreversible (relapse-free) growth inhibition in vitro was carried out as described elsewhere [14]. The AC_cure_ is the absolute concentration required to achieve sterile cure under in vitro conditions with incubation of compound for 24, 48 and 72 h, respectively. 

All experiments had two technical replicates and three biological replicates. Appropriate statistical tests were used for evaluating significance, and mean ± SEM was represented.

### 2.5. In Vivo Pharmacokinetic (PK) Analysis 

Determination of intravenous (i.v.) and per oral (p.o.) pharmacokinetics (PK) were carried out by using male BALB/c mice. For i.v. PK studies, compounds were formulated at a concentration of 2.5 mg/mL in 75% PEG300 and 25% D5W (5% dextrose in distilled water). To avoid any granular material, the solution was filtered, using a 0.45 μm syringe filter. The filtered solutions were dosed intravenously to mice via the lateral tail vein, at 5 mg/kg, with a dosing volume of 2 mL/kg. For all the p.o. PK studies, compounds were resuspended in 0.5% *v*/*v* methyl cellulose in 0.5 % *v*/*v* tween 80 solution. For 20 mg/kg dosing, 200 µL of compounds was administered orally to mice. Six blood samples of 50 μL each were collected serially from each animal, up to 24 h after dosing. Blood samples were collected into heparin microtainers and centrifuged, and then plasma was separated and frozen until analysis. Plasma samples (20 μL) were extracted with acetonitrile:methanol (3:1) containing internal standard. The samples were vortexed and then centrifuged in an Eppendorf centrifuge 5810R, at a setting of 4000 rpm, for 5 min, at 4 °C. The supernatant was transferred to a 96-well analysis plate and analyzed, using optimized LC/MS/MS conditions. For every experiment, 3 mice were used per compound, per dose. The plasma concentration-time profile was obtained by plotting the mean value from the three animals at each time point. Various PK parameters, such as C_max_ (maximum concentration), area under curve (AUC) and oral bioavailability (F) for compounds, were calculated by non-compartmental regression analysis, using an in-house fitting program developed at GNF [8]. All the in-life studies were carried out under protocols approved by the Animal Care and Use Committee (IACUC), following animal ethics guidelines of GNF.

For measurement of the brain-to-plasma drug concentration ratio, mice were dosed intravenously with 1 mg/kg compounds. Mice were euthanized at 5 and 60 min post-dosing, and blood and brains were collected. The compound concentrations in plasma and brains were measured, following the protocol described above.

### 2.6. Hemolymphatic Mouse Model (Stage 1 HAT Efficacy Model)

The *T. b. brucei* STIB795 acute mouse model mimics the hemolymphatic stage of the sleeping sickness disease. We used six female NMRI mice per experimental group, divided into two cages. Heparinized blood from a donor mouse with approximately 5 × 10^6^ per mL parasitemia was suspended in phosphate saline glucose (PSG), to obtain a parasite suspension of 1 × 10^5^ per mL. Each mouse was injected with 0.25 mL of parasite suspension (10^4^ bloodstream forms of *T. b. brucei* STIB795) intraperitoneally. All compounds were formulated in 0.5% methylcellulose and 0.5% Tween80. Three days post-infection, test compounds were administered orally on four consecutive days, in a volume of 100 µL/10 g. Three mice served as infected–untreated controls. The control mice were not injected with the vehicle, because we have established in our labs that this vehicle does not affect parasitemia or the mice. Until 31 days post-infection, parasitemia was monitored microscopically by tail-blood examination twice a week. Mice were considered cured when there was no parasitemia detected in the tail blood. All the results from the individual experiments were reported as number of mice cured over total number of mice treated. A Kaplan–Meier plot was used for representing the number of mice cured at different treatment doses.

In vivo efficacy studies in mice were conducted at the Swiss Tropical and Public Health Institute (Basel) (License number 2813), according to the rules and regulations for the protection of animal rights (“Tierschutzverordnung”) of the Swiss “Bundesamt für Veterinärwesen”. They were approved by the veterinary office of Canton Basel-Stadt, Switzerland.

### 2.7. Meningocephalic Mouse Model (Stage 2 HAT Efficacy Model)

The GVR35 mouse CNS model mimics the second (meningoencephalic) stage of African trypanosomiasis. Female CD1 mice (~8 weeks old, from Charles River) were injected intraperitoneally (i.p.) with 3 × 10^4^ *T. b. brucei* (GVR35-VSL2) bloodstream-form parasites [15]. As controls, a group of three untreated mice and another group of three mice treated with diminazene aceturate (DA) were included. The DA is a known anti-trypanosomal compound, which lacks brain penetration; hence, they clear only the blood parasitemia, leaving behind the parasites in brain. Mice treated with DA usually relapse after 42 days post-infection. Groups of six infected mice were dosed with TP compounds by oral gavage, once daily, from day 21 or 22 post-infection, for seven days. 

Blood parasitemia was quantified weekly from day 21/22, by microscopy of blood from the tail vein. Mice were imaged for bioluminescence using an in vivo imaging system (IVIS) prior to treatment on day 21/22, and weekly after treatment, as described previously [8,15]. Briefly, mice, in groups of three, were injected i.p. with 150 mg of D-luciferin (Promega) per kilogram body weight in PBS and imaged after 10 min, using an IVIS Spectrum (PerkinElmer, Waltham, Massachusetts MA, USA). Living Image Software (PerkinElmer) was used for acquisition and analysis of bioluminescence imaging data. Bioluminescence in the same rectangular region of interest (ROI) on whole-body mouse images was quantified and is shown in total flux (photons per second). Images were cropped to the ROIs and composites of images from representative mice are shown. Uncured mice were euthanized within 1 or 2 days of parasite detection in the blood. Cured mice were euthanized between day 92 and 101 post-infection. All the results from the individual experiments were reported as number of mice cured over total number of mice treated. A Kaplan–Meier plot was used for representing the number of mice cured at different treatment doses.

All animal protocols and procedures were reviewed and approved by the UK Home Office (Project License PPL60/4442 entitled “Molecular Genetics of Trypanosomes and Leishmania”) and University of York and University of Glasgow Ethics Committees, and was done in accordance with the Animals (Scientific Procedures) Act 1986 (ASPA).

## 3. Results

### 3.1. Extended Characterization of TP Series of Kinetoplastid Proteasome Inhibitors for Treatment of Human African Trypanosomiasis 

Previously, we described our efforts to identify novel chemotypes for the treatment of HAT. These efforts led the identification of GNF6702, a prototypical pan-kinetoplastid inhibitor that was efficacious in the mouse models for all three kinetoplastid diseases [8]. 

**Figure 1:**
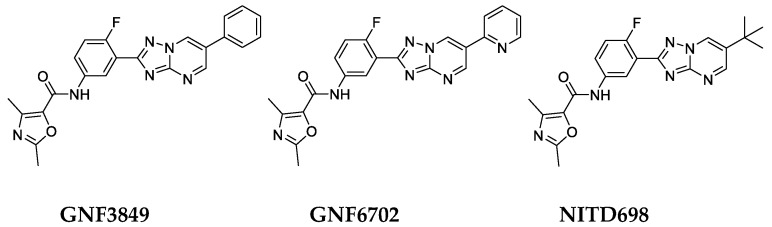
Chemical structure of the triazolopyrimidine class of inhibitors.

All the three TP compounds (GNF3849, GNF6702 and NITD689) showed potent growth inhibition (EC_50_ < 70 nM) against *T. b. brucei* with varying cytotoxicity profile (Figure 1). Further evaluation of triazolopyrimidine analogues for the optimal blood–brain barrier penetration properties, such as lipophilicity (cLogP) and polar surface area, led to prioritization of NITD689, in addition to GNF3849 and GNF6702. All three compounds exhibited good membrane permeability, low mouse liver microsomal clearance and moderate lipophilicity (Figure 1). While GNF3849 had favorable potency against *T. brucei*, it was cytotoxic against HepG2 cells. Moreover, it also suffered from poor solubility and high plasma protein binding, indicating that higher total exposure might be needed to observe efficacy in in vivo. As reported previously [8], replacement of a phenyl group of GNF3849 with a pyridine moiety (GNF6702) improved solubility, lowered PPB and also created a molecule with a better cytotoxicity. Medicinal chemistry optimization by increasing SP^3^ fraction within the molecule by replacing pyridine group with a tertiary butyl group led to the identification of NITD689. The NITD689 showed improved solubility, reduced plasma protein binding and retained non-cytotoxic profile, with 30 nM potency against *T. b. brucei* (Figure 1 and Table 1). 

### 3.2. Biological Profiling of the TP Class of Inhibitors

**Figure 2:**
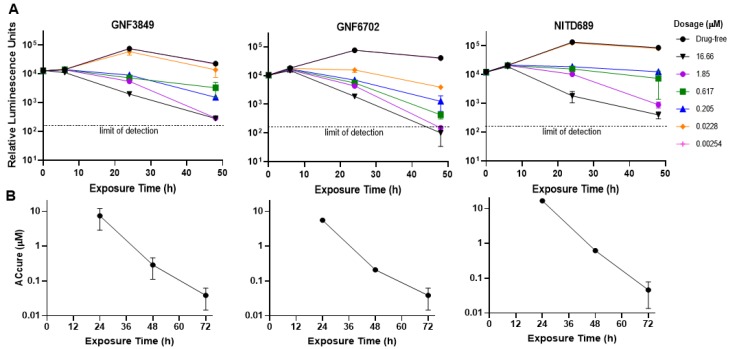
Biological characterization of the TP class of compounds. (**A**) Time-to-kill profile indicating concentration-time dependent kill. (**B**) To achieve sterile cure without relapse under in vitro conditions. All experiments were carried out three times, independently, and mean ± SEM was plotted.

Triazolopyrimidines were evaluated for their potential to kill parasites under in vitro conditions. Time-to-kill assessment showed that all the three compounds were trypanocidal. At an early time point (6 h post exposure), no significant cidality was seen compared to the untreated control, even at the highest concentration tested (16.7 µM). However, at 24 and 48 h post-incubation, with compounds, significant kill was evident, suggesting this class of inhibitors requires 24 h in order to show cidality. All three compounds showed concentration and time-dependent lethality (Figure 2A). 

In order to evaluate the ability of TP compounds to kill all parasites without recrudescence (sterile cidality), we carried out drug wash-out assays. All the three TP compounds achieved sterile cure with ACcure (Absolute concentration of drug required to kill parasites without relapse) values of <100 nM, after a 72-h compound treatment and subsequent wash-out. The ACcure values also decreased as the time of exposure increased, indicating exposure-driven sterile cure (Figure 2B).

### 3.3. The TP Class of Inhibitors Are Active against Clinical Isolates

GNF3849 and GNF6702 were also tested for their ability to inhibit growth of clinical isolates of *T. b. gambiense* and *T. b. rhodesiense*. Our compounds showed single-digit nM potency against all strains tested. The compounds were also active against pentamidine- and melarsoprol-resistant strains of *T. b. rhodesiense*, as well as *T. b. brucei* (Table 2), suggesting a distinct mechanism of action, at least in terms of drug uptake, since resistance to these drugs relates to loss of transporters specific for their uptake [16,17].

### 3.4. The TP Class of Compounds Are Proteasome Inhibitors

The TP class of inhibitors is active against both *T. cruzi* and *T. brucei.* Resistance selection in *T. brucei* proved difficult; hence, we generated *T. cruzi* resistant isolates, using a classical resistant mutant generation approach. Whole-genome sequencing identified single nucleotide polymorphisms in F24L and I29M of β4 subunit of 20S proteasome. Further validation of the proteasome as a target for the TP class of compounds has been described elsewhere [8]. *T. brucei* parasites modified to express an F24L mutant β4 subunit of the 20S proteasome were previously shown to be resistant to the parent proteasome inhibitor. Here, we used the same strain to demonstrate its resistance to the new TP compounds, confirming that they, too, target the proteasome (Table 3). Meanwhile, bortezomib, a known chymotrypsin proteasome inhibitor, was equipotent against both wild-type and F24L mutant of *T. brucei*, suggesting that bortezomib and TP compounds interact differently by binding into different pockets within 20S proteasome.

### 3.5. In Vivo Pharmacokinetic Properties of TP Class of Compounds

In vivo mice PK profiling was carried out, using intravenous and oral routes (Table 4). Following intravenous administration, all three TP compounds displayed a moderate volume of distribution (V_ss_: 1.2 to 1.5 L/kg), low total systemic clearance (2.5 to 15% of hepatic blood flow) and moderate-to-long elimination half-life (1.6 to 7 h). Following oral administration at 20 mg/kg, all three TP compounds showed good oral exposure, with bioavailability ranging from 34% to 100%. The dose-normalized total exposure was highest for GNF6702 in terms of AUC, followed by GNF3849 and NITD689. Although NITD689 had the lowest exposure in terms of total concentration, it had the highest free C_max_ and better free AUC compared to GNF3849, due to low plasma protein binding (Figure 1 and Table 4). 

### 3.6. TP Class of Compounds Is Efficacious against Hemolymphatic Infection in a Stage 1 HAT Mouse Model

**Figure 3:**
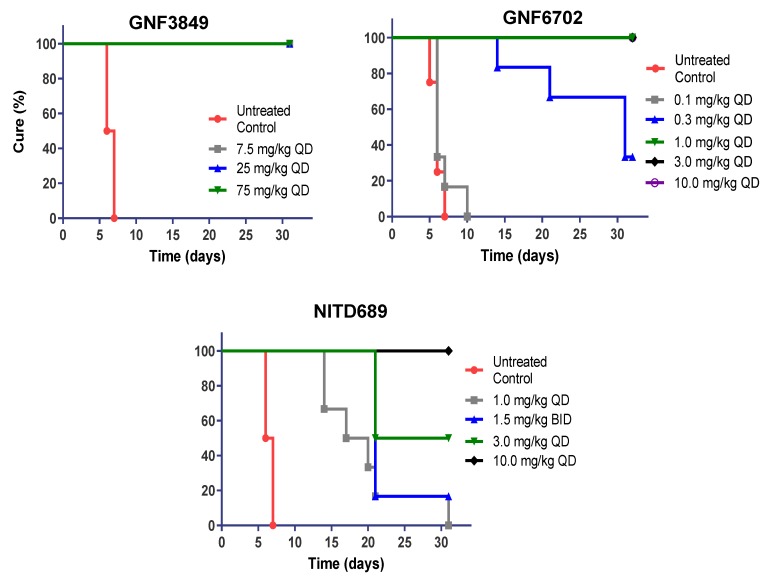
In vivo efficacy of GNF3849, GNF6702 and NITD689 in a HAT hemolymphatic mouse model. Six mice each were orally treated for four days, with varying doses of compounds, three days post-infection. Mice were monitored for 30 days post-infection, and cure plot (Kaplan–Meier plot) showing percentage of animals cured over time are shown.

Since all three TP compounds showed promising in vivo PK properties in mice, they were evaluated for their ability to achieve cure in a mouse model of stage 1 (hemolymphatic) disease. GNF3849 achieved complete cure at all doses (7.5, 25 and 75 mg/kg, once daily, for four days). The other two compounds, GNF6702 and NITD689, showed dose-dependent cure, with increasing doses showing better cure (Figure 3). Minimum efficacious doses for GNF6702 and NITD689 were 1 and 10 mg/kg QD dosing for four days, respectively (Table 5). 

### 3.7. Assessment of Brain Permeability, Tissue Binding and Partitioning of TP Compounds

In order to evaluate potential of brain permeability, all three compounds were tested in an MDR1-MDCK permeability assay. Compounds with better A to B permeability and reduced efflux ratio have good potential to reach high concentrations in the brain. Those that have a high efflux ratio are generally substrates for Pgp transporters, thereby having a higher propensity to be excluded from the brain. All three compounds had reasonable permeability and an efflux ratio of <3.5 (Table 6). To assess directly the TP compounds’ ability to penetrate brain, mice were dosed with compound, and plasma and brains were collected after 5 and 60 min following intravenous dosing. GNF3849 had the highest brain-to-plasma ratio (B/P ratio), followed by GNF6702 and NITD689 (Table 6). Although GNF3849 had a better B/P ratio based on total concentration, it had high brain tissue protein binding (>99%), whereas GNF6702 and NITD689 had lower brain tissue binding (94.7% and 96.5%, respectively). This implies that the free concentration available at the site of infection could be lower for GNF3849 compared to the other two TP compounds. 

### 3.8. TP Class Compounds Are Efficacious against Meningoencephalic Infection in HAT Mouse Model

**Figure 4:**
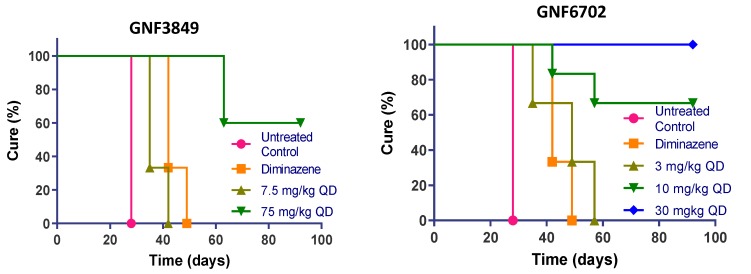

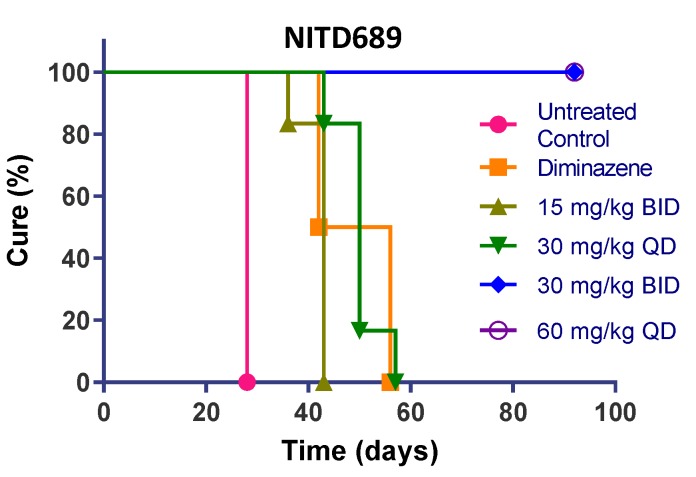
In vivo efficacy of GNF3849, GNF6702 and NITD689 in HAT meningoencephalic mouse model. Six mice each were orally treated for seven days, with varying doses compounds, 21or 22 days post-infection. Mice were monitored for 92–94 days post-infection, and cure plots (Kaplan–Meier plots) showing percentage of animals cured over time are shown. Three mice each were dosed with vehicle control and diminazene aceturate. Note the early parasite recrudescence in mice treated with diminazene aceturate.

**Figure 5:**
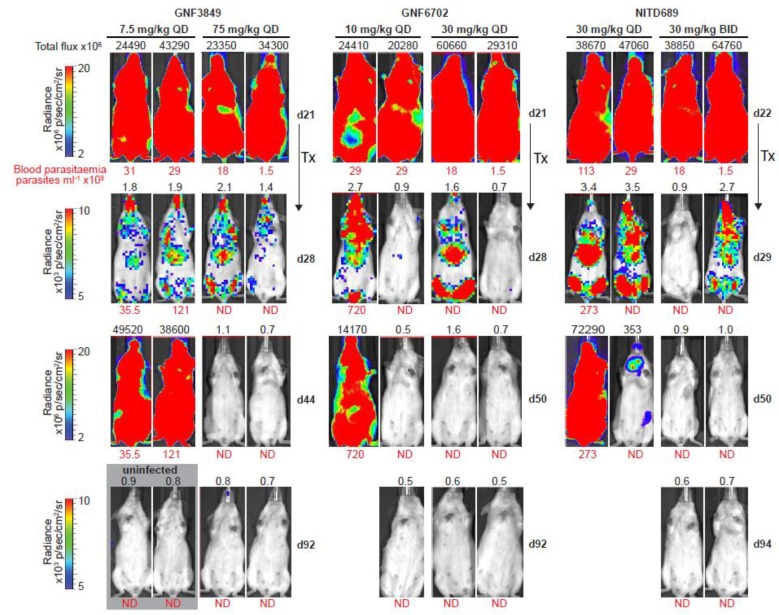
Bioluminescence imaging of mice infected with *T. b. brucei*. Dose-dependent clearance of parasites from triazolopyrimidine class of inhibitors. In vivo quantification of bioluminescent *T. b. brucei* (GVR35–VSL2) in infected mice before and after treatment; day 21/22, start of treatment; day 28/29, 24 h after last dose; day 44/50 and day 92/94, parasite recrudescence or cure in mice treated with GNF3849, GNF6702 and NITD689 (images of two representative mice from a total of six mice are shown). Blood parasitemia (in parasites/mL, red font below image) and whole mouse total flux (in photons per second, black font above image) values of each animal are shown; QD, once daily; BID, twice daily; N.D., not detectable; Tx, treatment. The same two representative mice are shown for all time points. Mice with detectable parasites were euthanized and are therefore not shown at day 92/94. Images from uninfected mice, aged-matched for day 21, that were collected independently, using the same acquisition settings, are shown in the gray box (two of three mice are shown).

Having demonstrated brain permeation, all three compounds were evaluated in a stage II (meningoencephalic) mouse model of HAT, with various doses (Figure 4). The recrudescence of bioluminescent parasites was monitored by using an in vivo imaging system over a period of three months (Figure 5). GNF6702, which had the best exposure and longest half-life, was dosed by oral gavage at 3, 10, 30 and 100 mg/kg, once daily. A dose-dependent increase in cure rate was noticed, with 30 and 100 mg/kg showing complete cure, and 10 mg/kg showing partial cure, with four out of six mice achieving relapse-free cure (Table 7). GNF3849 was the next best molecule, with reasonable exposure (~2.5 fold less than GNF6702) and a long half-life of 6.5 hours. Hence, they were dosed at 7.5 and 75 mg/kg, once daily. GNF3849 failed to achieve complete cure at either dose with 75 mg/kg, showing only 50% cure. GNF3849 had high plasma protein (>99%) and brain tissue binding (>99%), which could have resulted in significantly lower free concentration of compounds required to achieve 100% relapse-free cure. 

NITD689 had promising physicochemical properties, better permeability and the lowest efflux ratio, although the exposure was lower than GNF6702 (Table 2, Table 4 and Table 6). NITD689 also had a moderate half-life of 1.6 h; hence, we adopted both a once-daily and twice-daily dosing schedule. Both doses of 15 mg/kg twice daily and 30 mg/kg once daily failed to cure, suggesting that the time and concentrations reached were not sufficient to kill all parasites in the brain. However, 30 mg/kg twice daily and 60 mg/kg once daily of NITD689 achieved complete cure, without relapse. 

## 4. Discussion

Several research groups have been working on the development of anti-trypanosomal compounds with a potential to treat HAT. The most significant challenge has been achieving brain penetration for chemical molecules, which is critical for killing brain stage parasites to achieve complete cure in stage 2 infection. For example, N-myristoyltransferase inhibitors have been shown to be potent trypanocides and curative of stage 1 models of the disease, but failed to achieve reasonable brain concentrations, thereby leading to failure to cure stage 2 models of HAT [18]. Attempts to screen compound libraries specifically inhibiting kinases [19] and proteases [20] also identified potent trypanocides which failed to achieve complete cure in mouse models, due to lack of adequate brain penetration. Our attempts to find novel trypanocides led to the identification of potent growth inhibitors. Further medicinal chemistry optimization of compounds led to brain-penetrant derivatives belonging to TP class (GNF6702 and NITD689) which completely cured both stages of infection. 

We had previously reported that the TP class of molecules are chymotrypsin proteasome inhibitors with pan-kinetoplastid activity [8]. All the compounds described in the current manuscript also inhibited chymotrypsin activity of the 20S proteasome in *T. brucei*. The *T. b. brucei* strain overexpressing F24L mutation in β4 subunit of 20S proteasome showed greater than 60-fold shift in growth inhibition concentration for the TP compounds, confirming on-target activity. Recently, Wyllie and co-workers also described proteasome inhibitors with the potential to treat visceral leishmaniasis [21]. The proteasome inhibitors described here demonstrate great potency against clinical isolates of *T. b. gambiense* (EC_50_ = < 10 nM), compared to fexinidazole (EC_50_ = 0.95–3.3 µM) [9] and acoziborole (EC_50_ = 0.18–1 µM) [14]. Our compounds also showed concentration and time-dependent cidality and relapse-free kill of all parasites in wash-out assays in vitro, at concentrations below 100 nM. These cidality properties are essential for achieving cure in HAT mouse models [22]. Extensive medicinal chemistry optimization helped in improving PK properties required to achieve moderate brain penetration, which proved essential to cure brain infection. Both GNF6702 and NITD689 completely cured a stage 2 infection in a HAT mouse model, at 30 and 60 mg/kg dose, respectively. In addition, the exposures reached in the animal models were higher than their respective ACcure concentrations required for sterilization in vitro. Furthermore, these doses were lower than the curative dose of fexinidazole [9] (200 mg/kg) and comparable to that of acoziborole (25 mg/kg) [14]. A detailed structure activity relationship of TP class of compounds against both *T. brucei* and *T. cruzi* has been described by Nagendar and co-workers [23]. Although, one of their compounds, compound **20**, had a brain-to-plasma ratio of 0.23, it was not profiled in the stage 2 HAT model, due to higher plasma protein binding (98.5%), which might affect free concentration in brain required for achieving stage 2 efficacy.

While other developments in the treatment of HAT have been very promising, the TP class of proteasome inhibitors has significant potential for further progress. Other than acoziborole, which is in phase II studies for HAT, the proteasome inhibitors described here are the most advanced compounds with drug-like properties. They not only have promising in vitro and in vivo potency in disease relevant HAT models, but also have favorable pharmacokinetic properties, with potential for further development. 

## Figures and Tables

**Table 1:** Biological, physicochemical and in vitro pharmacokinetic properties of the triazolopyrimidine inhibitors.

Assays/Properties *	GNF3849 ^#^	GNF6702 ^#^	NITD689 ^$^
*T. b. brucei* EC_50_ (nM)	22 ± 4	70 ± 3	30 ± 4
HepG2 CC_50_ (µM)	1.1 ± 0.2	>20	>20
Solubility pH 6.8 (g/L)	<0.002	0.009	0.071
LogD/Mol Wt/PSA	3.31/428/98	2.32/429/103	3.05/408/98
MPO	3.4	3.7	3.6
PAMPA Permeability (% FA)	96	92	99.1
Mouse liver microsomal clearance (µL/min/mg)	15.2	34.1	7.41
Mouse plasma protein binding (%)	>99	95.1	90.5

Note: All EC_50_ and CC_50_ values correspond to mean ± SEM (n = 4 biological replicates); * LogD: measured octanol water co-efficient; Mol wt: molecular weight; PSA: polar surface area; MPO: multi-parametric optimization; PAMPA: parallel artificial membrane permeability assay; FA: fraction absorbed. **^#^** Khare et al., Nature 2016 [8]; ^$^ patent US 2019/0000852 A1.

**Table 2:** Growth inhibitory profile of the triazolopyrimidine class of compounds against various parasite strains.

Parasite	Strain	GNF3849	GNF6702	Melarsoprol	Pentamidine
*T. b. gambiense* (EC_50_ in nM)	STIB930	4.5 ± 1	3.7 ± 2	7.1 ± 2.7	1.4 ± 0.9
	K048	4.8 ± 1.1	3.5 ± 1.4	10.2 ± 1.9	33.9 ± 16
	R130	1.9 ± 1	2.3 ± 1.1	7.7 ± 3.1	13.5 ± 4
*T. b. rhodesiense* (EC_50_ in nM)	STIB900	1.4 ± 1	1.6 ± 0.9	3.9 ± 2	2.1 ± 0.7
	STIB900 Pent^R^	1.7 ± 1	1.2 ± 0.3	46.8 ± 19	203 ± 54
	STIB900 Mel^R^	0.6 ± 3	0.7 ± 0.2	95.5 ± 24	217 ± 86
*T. b. brucei* (EC_50_ in nM)	BS221	1.9 ± 0.8	0.9 ± 0.3	4.7 ± 1.8	0.3 ± 0.1
	BS221 (P2 KO)	4.0 ± 2.5	3.5 ± 1.2	37.7 ± 14.8	3.1 ± 0.4
	STIB950	0.9 ± 0.2	1.2 ± 0.5	16.9 ± 8.8	0.5 ± 0.2

Note: All EC_50_ values correspond to mean ± SD (n = 3 biological replicates).

**Table 3:** Mutations in F24L in the 20S proteasome β4 subunit confers resistance to TP class of compounds.

Strain	*T. brucei* PSMB4^WT^ (EC_50_ = nM)	*T. brucei* PSMB4^F24L^ (EC_50_ = nM)
GNF3849	10 ± 3	2200 ± 20
GNF6702	18 ± 1.8	1200 ± 13
NITD689	20 ± 4	1900 ± 20
Bortezomib	0.94 ± 0.05	1.1 ± 0.26

Note: All EC_50_ values correspond to mean ± SEM (n = 3 biological replicates); PSMB^WT^: *T. brucei* ectopically expressing wild-type copy of 20S proteasome β4 subunit; PSMB^F24L^: *T. brucei* ectopically expressing F24L mutant copy of 20S proteasome β4 subunit.

**Table 4:** In vivo pharmacokinetics properties of the TP class of compounds.

Parameters	Units	GNF3849	GNF6702	NITD689
**I.V. PK**				
Dose	mg/kg	5	5	5
V_ss_	L/kg	1.2	1.2	1.5
CL	mL/min/kg	2.46	2.26	13.48
T_1/2_	H	6.5	7.0	1.6
**P.O. PK**				
Dose	mg/kg	20	20	20
C_max_	nM	7160 (<71.6)	13,668 (669.7)	12,170 (1156.2)
T_max_	H	3	7.67	0.5
AUC	nM*h	107,510 (<1075.1)	271,489 (13,303)	89,692 (8235.7)
F	%	34	79	100

Note: I.V. PK: intravenous pharmacokinetics in mouse; P.O. PK: per oral pharmacokinetics in mouse; V_ss_: Volume of distribution at steady state; CL: total systemic clearance; T_1/2_: Elimination half-life; C_max_: maximum concentration reached in blood, values in parenthesis represent free fraction; T_max_: time to reach maximum concentration; AUC: exposure between 0 to infinity, values in parenthesis represent free fraction; F: oral bioavailability.

**Table 5:** In vivo efficacy of GNF3849, GNF6702 and NITD689 in a HAT hemolymphatic mouse model.

Compound ID	Dose (mg/kg)	Dose Frequency	Mice Cured/Total	Mean day of Relapse	% Cured
**GNF3849**	7.5	QD	6/6	>31	100
	25	QD	6/6	>31	100
	75	QD	6/6	>31	100
**GNF6702**	0.1	QD	0/6	6.83	0
	0.3	QD	2/6	19	33.3
	1	QD	6/6	>31	100
	3	QD	6/6	>31	100
	10	QD	6/6	>31	100
**NITD689**	1	QD	0/6	19.5	0
	1.5	BID	1/6	21	16.7
	3	QD	3/6	21	50
	10	QD	6/6	>31	100

NOTE: Mean day of relapse refers to days post infection; QD = once daily; BID = twice daily.

**Table 6:** Assessment of brain permeability, tissue binding and partitioning of TP compounds.

Compound ID	BTB (%)	MDR1-MDCK		Efflux Ratio	B/P Ratio	
		A–B	B–A	B–A/A–B	5 min	60 min
**GNF3849**	>99	4.5	14.1	3.2	0.22	0.28
**GNF6702**	94.7	8.3	22.1	2.7	0.13	0.17
**NITD689**	96.5	32.6	29.2	0.9	0.12	0.12

Note: BTB: rat brain tissue binding; MDR1-MDCK = Multi Drug Resistant 1 overexpressing Madin–Darby canine kidney cells; A–B = Apical to Basolateral; B–A = Basolateral to Apical; B/P ratio = brain-to-plasma ratio.

**Table 7:** In vivo efficacy of GNF3849, GNF6702 and NITD689 in a HAT meningoencephalic mice model.

Compound ID	Dose (mg/kg)	Dose Frequency	Mice Cured/Total	Mean Day of Relapse	% Cured
**GNF3849**	7.5	QD	0/6	37	0
	75	QD	3/6	60 (>92 *)	50
**GNF6702**	3	QD	0/6	47	0
	10	QD	2/6	50 (>92 *)	66
	30	QD	6/6	>92	100
	100	QD	6/6	>92	100
**NITD689**	15	BID	0/6	42	0
	30	QD	0/6	50	0
	30	BID	6/6	>94	100
	60	QD	6/6	>94	100
**Diminazene**	40	QD	0/3	42	0

NOTE: Mean day of relapse refers to days post infection; * mean values shown are for the mouse which relapsed; values in parenthesis are for mice which did not relapse; QD = once daily; BID = twice daily.
